# Mapping the bioinformatics resource landscape: an analysis of LibGuides across Canadian universities

**DOI:** 10.29173/jchla29878

**Published:** 2025-12-01

**Authors:** Grailing Anthonisen, Lisa Banks

**Affiliations:** 1 MA, (MISt in progress), McGill University, University of New Brunswick – Saint John, Montreal, QC; 2 MA, MISt, McGill University, Acting Collections Management Librarian, New Brunswick Public Library Service, Fredericton, NB

## Abstract

**Introduction:**

The initial introduction of LibGuides created a seismic shift in bioinformatic information dissemination. This study reviewed bioinformatics LibGuides from Canadian universities to establish a consistent overlap of frequently recommended materials that would spell out a canon of bioinformatics resources.

**Methods:**

We undertook a manual review of the 11 bioinformatics-specific LibGuides created by Canadian university libraries.

**Results:**

We found four (n=4) LibGuides focused solely on bioinformatics and seven (n=7) guides with subsections dedicated to bioinformatics. Overall, there were 566 resources distributed across 11 LibGuides, with little overlap across guides. Most (n=440) were distinct resources. The most common resource type was database and the most frequently appearing resources (n=5) were BLAST Database, Protein Data Bank, and PubMed.

**Discussion:**

We identified clear variations in the intended audience for these subject guides, as well as commonalities across all 11. The diversity of listed resources reflects the diversity and interdisciplinary nature of bioinformatics. Despite this variety of resources, we observed a uniformity in those resources’ funding or hosting, as they are often American in origin. This now represents a concern for librarians and other information professionals when it comes to using and teaching bioinformatics resources.

## Introduction

With its earliest beginnings as a field traced to the 1960s [1], bioinformatics has undergone an explosive transformation in the intervening decades. As an interdisciplinary field at the intersection of “biological science, computer science, and information science” [1], bioinformatics offers a near-overwhelming plethora of resources. Bioinformatics resources, outreach, and subject-specific information dissemination were once primarily the domain of in-person library workshops [2], necessitating attendance and engagement at a fixed time, in a synchronous format. This has since changed drastically, in part due to the introduction and proliferation of online instruction. The rise of the internet as a primary mode of communication has also seen the rise of asynchronous learning resources, including subject-specific resources hosted by universities—like those created by academic librarians and information professionals, for academic libraries. Earlier reliance on either in-person library workshops or print subject guides [4] has changed drastically due to the adoption of electronic content management systems (CMS), as well as the COVID-19 pandemic more recently.

These CMS, with their ease-of-use (no coding experience is required of the information professional creating the guide), offer a clear, straightforward avenue for information dissemination. One such CMS is SpringShare’s LibGuides platform, first launched in 2007 [3]. LibGuides, which markets itself as “the most popular content management and curation platform for libraries” [5], allows users to discover resources on their own terms and in their own time. As a key component of academic libraries [6,7], these resources may include database search strategies and tutorials, information on the specific library’s discovery layer, link-outs to subject-specific resources, or any other resources deemed relevant by the information professional creating the guide. Guides may be either subject-specific or course-specific. While previous research has examined in-person, library-based bioinformatics workshops as a means of information dissemination [2], the implementation of LibGuides has meant a seismic shift in information dissemination. While the literature variously refers to these guides and similar wayfinding resources with a variety of terminologies [9], we use the term LibGuides in this paper to refer to the SpringShare platform. Our analysis focused *specifically* on SpringShare’s LibGuides, as mention of the specific platform informed our search strategy (discussed below).

Geer observed in 2006 that “the rapid growth in the number and complexity of bioinformatics resources seems to outpace most researchers’ ability to keep up with the fast-moving field” [8]. In a similar study that same year at Purdue University, researchers “regularly communicated that they greatly needed help with bioinformatics resources” [12]. Two decades on, and with information being generated ever faster in the internet age alongside the decreased cost of sequencing, this remains unchanged—there is more data produced, and at a greater scale. Health librarians, with their expertise in information organization, curation, dissemination, and explanation, offer one way to help meet the needs of this fast-moving field. As Geer observed, one method to ensure ease-of-access to bioinformatics resources may include web portals or other online resources [8]—we view LibGuides as just such a resource.

While the relationship between health professions and librarianship has been studied [13], the relationship between bioinformatics resources and their dissemination by information professionals via asynchronous platforms such as LibGuides, requires further examination. The initial goal of this project was to establish a consistent overlap of commonly recommended materials that would spell out a canon of bioinformatics resources. We believed a manual review of bioinformatics-related LibGuides from Canadian universities would reveal clear information overlap between each library’s—and thus university’s—chosen resources. To our surprise, we found that this was not the case, and so we sought to figure out why. Previous research analyzing LibGuides’ usage and efficacy has often coupled content analysis of the guides themselves with qualitative surveys [9,15], but this was beyond the scope of our analysis.

### Research questions

To guide our analysis of bioinformatics-related LibGuides, we developed the following research questions:

1. How does the interdisciplinarity of bioinformatics reflect the resources selected for LibGuides?
2. What common resources appear across institutions’ LibGuides? What divergences are there in suggested resources?
3. What are the implications of the resource providers and the sources, both those that appear frequently and infrequently across LibGuides?

Searches in Library and Information Science Abstracts (LISA) and Library, Information Science and Technology Abstracts (LISTA) for bioinformatics LibGuides returned few relevant results. This suggests that a thorough overview of Canadian universities’ bioinformatics LibGuides is necessary in an ever-evolving information landscape. Though certainly not the first analysis of LibGuides in academic libraries [3,16], this is the first to our knowledge to focus specifically on bioinformatics-related LibGuides in a Canadian university context.

## Methods

To develop our search strategy, between March and April of 2025 we drew from a list of 81 public universities in Canada [17], both anglophone and francophone. Due to potential variation across library websites, we used search engines to identify relevant LibGuides. For anglophone institutions, we searched “[*name of university*] libguide bioinformatics” or “[*name of university*] ‘libguide’ bioinformatics” on DuckDuckGo and Google.com. For francophone universities, we adjusted our search terms to “[*name of university*] libguide bio-informatique” and “[*name of university*] guide de recherche bio-informatique” on DuckDuckGo and Google.com. As the search results typically brought us to the LibGuide itself or the library’s LibGuide home page, this was an efficient approach to searching 81 university libraries.

To offer preliminary answers to our queries, we undertook a manual review of all 11 Canadian university LibGuides which cited bioinformatics in either the guide’s title or as a subsection of a broader-topic LibGuide. As our focus was on Canadian universities, we did not include special libraries or hospital libraries which mentioned bioinformatics. Our inclusion criteria required the guides to list “bioinformatics” in either the title of the guide or in one of its subsection titles. Other types of informatics that may overlap with bioinformatics, such as health informatics, were excluded unless bioinformatics was specifically cited.

**Fig. 1:**
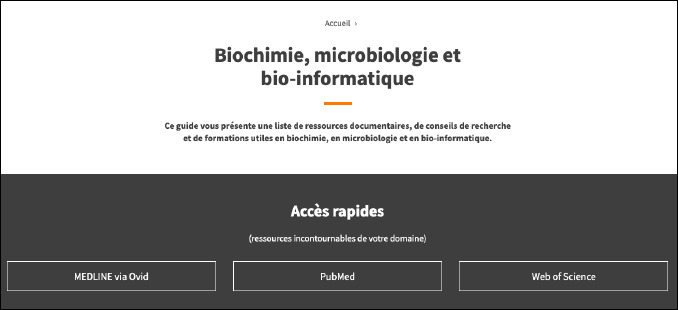
Demonstrates the top of Laval’s LibGuide that highlights MEDLINE, PubMed, and Web of Science

**Fig. 2:**
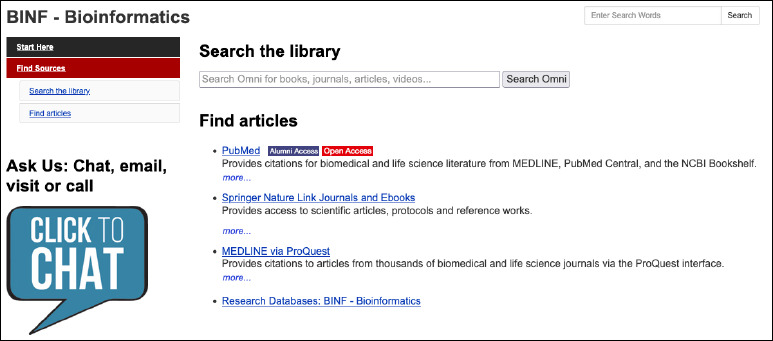
Shows Guelph’s to-the-point choice of resources

Once we identified the 11 LibGuides that met our inclusion criteria, we manually reviewed them. Data collection involved saving each resource’s web link, title, the guide it belonged to, the provider or host of the resource, and any other relevant notes, to an Excel sheet. In a separate sheet, we noted subsection titles which we then categorized via descriptive analysis according to their stated uses. We categorized resources according to their types and drew from the language used by the guides to do so. For example, while PubMed is an interface, this paper will reflect the language of the guides, which refer to PubMed as a database. Similarly, while MEDLINE is a subset of PubMed, we differentiate them as they are listed as separate resources in several guides, such as Laval and Guelph’s (Figures 1 and 2). Another indicator for categorization was the resources’ title or heading on its landing page. This means that we differentiated National Center for Biotechnology Information (NCBI) databases from National Library of Medicine (NLM) resources or National Institutes of Health (NIH) webpages. Using the JMP Student Edition 18 statistical program, we coded and analyzed the data to derive quantitative analysis, mainly general descriptive statistics relating to the data, to support our analysis.

## Results

We found four (n=4) LibGuides focused solely on bioinformatics and seven (n=7) guides with subsections dedicated to bioinformatics (Table 1). McGill University (McGill), University of Guelph (Guelph), Université Laval (Laval), and York University (York) had full guides. Simon Fraser University (SFU), University of Calgary (Calgary), Université de Montréal (UdeM), University of Manitoba (Manitoba), University of Toronto (Toronto), and University of Winnipeg (Winnipeg) each had subsections of larger guides dedicated to bioinformatics, with UdeM having different subsections on two different guides. The larger LibGuide topics were biology (Winnipeg), informatics (referencing theoretical bioinformatics; UdeM), biochemistry and molecular medicine (referencing practical bioinformatics; UdeM), and basic medical sciences (Manitoba). The Toronto subsection was part of a class on plant biology as part of the biology department. We also included the LibGuides for the “Centre for Career and Personal Development Degree Profiles”, which provides an outline of potential career paths aligned with different degrees provided by Calgary, and molecular biology and biochemistry, as was the case for SFU. Most guides were updated in 2025 (n=5) or 2024 (n=2), with the exception of one not updated since 2020. Neither the UdeM nor the Laval guides provided update information.

**Table 1:** LibGuide focus and last known update

University	LibGuide Focus	Sole focus of the guide or subsection of a guide	Date of update
McGill University	Bioinformatics	Sole focus of the guide	3 March 2025
Simon Fraser University	Molecular Biology and Biochemistry	Subsection of a guide	22 December 2020
Université Laval	Biochemistry, microbiology and bioinformatics	Sole focus of the guide	Unknown
Université de Montréal (Bio-informatique pratique)	Biochemistry and molecular medicine	Subsection of a guide	Unknown
Université de Montréal (Bio-informatique théorique)	Informatics	Subsection of a guide	Unknown
University of Calgary	Centre for Career and Personal Development Degree Profiles	Subsection of a guide	22 April 2025
University of Guelph	Bioinformatics	Sole focus of the guide	23 April 2025
University of Manitoba	Basic Medical Sciences	Subsection of a guide	14 April 2025
University of Toronto	BIO353H5: Plant Development	Subsection of a guide	2 April 2025
University of Winnipeg	Biology	Subsection of a guide	18 September 2024
York University	Bioinformatics	Sole focus of the guide	19 December 2024

Most guides were organized around finding literature, with section titles like “Find articles” (n=2; UdeM theoretical and Guelph), or “Books and E-books” (n=4; Laval, UdeM theoretical, Manitoba, SFU). McGill’s guide was organized thematically and around databases (i.e., databases and tools were organized under headings like “Genes, nucleotides and genomes” and “Proteins and proteomes”). Winnipeg titled its section with the generic “Sources,” while exclusively listing gene databases and a tool annotated with a description of its ability to search gene data.

Overall, there were 566 resources distributed across 11 LibGuides (Table 2).

**Table 2:** Resource numbers per LibGuide and overall percentage of the total resources across all LibGuides

University	Number of resources per LibGuide	Overall percentage of thetotal resources across all LibGuides
McGill University	61	10.8%
Simon Fraser University	9	1.6%
Université Laval	196	34.6%
Université de Montréal (Bio-informatique pratique)	113	20%
Université de Montréal (Bio-informatique théorique)	48	8.5%
University of Calgary	20	3.5%
University of Guelph	5	0.9%
University of Manitoba	16	2.8%
University of Toronto	3	0.5%
University of Winnipeg	3	0.5%
York University	92	16.25%

There was little overlap of resources across guides, with 440 unique resources linking to distinct resources, with some LibGuides listing a resource multiple times and other LibGuides not listing it at all^2^. The most commonly used resources were databases, with BLAST (Basic Local Alignment Search Tool) Database, Protein Data Bank (PDB), and PubMed each appearing on five (n=5) different institutions’ LibGuides. Multiple resources appeared in four (n=4) different LibGuides: FlyBase, GenBank, NCBI Tutorials, Nucleic Acids Research web servers, and UniProt—several of which are also databases. Another seven databases appear in three different LibGuides (Table 3). Organism-specific databases (FlyBase, WormBase, Saccharomyces Genome Database, TAIR, ZIN), are well-represented, reflecting the specialized nature of biological research across different model organisms. The most common resources (linked 3-5 times) appeared most frequently on McGill, Laval, and UdeM’s LibGuides. The most overlap occurred among databases. However, in most cases, resources with any overlap were referenced in only two different LibGuides; 32 out of the 49 total repeat resources were referenced twice (Table 3).

**Table 3:** Resources used more than once across LibGuides, with 49 resources repeated a total of 126 times. The table is organized according to resource numbers (descending) and then alphabetically within types.

Resource type	Resource name	Number of times resource appears	LibGuideswhere the resource appears
Database	BLAST (including tutorial)	5	McGill University Université Laval Université de Montréal (Bio-informatique pratique) University of Winnipeg York University (included a tutorial as well)
	Protein Data Bank	5	McGill University Université Laval Université de Montréal (Bio-informatique pratique) Université de Montréal (Bio-informatique théorique) York University
	PubMed	5	McGill University Université Laval University of Guelph Université de Montréal (Bio-informatique théorique) York University
	FlyBase	4	McGill University Université Laval Université de Montréal (Bio-informatique pratique) York University
	GenBank	4	Université Laval Université de Montréal (Bio-informatique pratique) Université de Montréal (Bio-informatique théorique) •York University
	UniProt	4	McGill University Université Laval Université de Montréal (Bio-informatiquepratique) York University
	Conserved Domains	3	McGill University Université de Montréal (Bio-informatiquepratique) York University
	European Bioinformatics Institute (including tutorials)	3	McGill University Université de Montréal (Bio-informatiquepratique) York University
	Ensembl (including tutorials)	3	McGill University Université Laval Université de Montréal (Bio-informatiquepratique)
	Gene	3	McGill University Université de Montréal (Bio-informatiquepratique) York University
	Genome	3	McGill University Université Laval Université de Montréal (Bio-informatiquepratique)
	Protein	3	McGill University Université Laval York University
	SciFinder	3	McGill University Université Laval Université de Montréal (Bio-informatiquethéorique)
	UCSC Genome Browser	3	McGill University Université de Montréal (Bio-informatiquepratique) University of Winnipeg
	ArrayExpress	2	McGill University Université de Montréal (Bio-informatique pratique)
	DNA Databank of Japan	2	McGill University Université de Montréal (Bio-informatiquethéorique)
	Entrez	2	University of Winnipeg York University
	European Nucleotide Archive	2	McGill University Université de Montréal (Bio-informatiquethéorique)
	Expasy	2	Université de Montréal (Bio-informatiquethéorique) York University
	Gene Expression Omnibus (GEO)	2	McGillUniversity Université de Montréal (Bio-informatiquepratique)
	MEDLINE	2	University of Guelph Université Laval
	Mouse Genome Informatics (MGI)	2	McGill University Université de Montréal (Bio-informatiquepratique)
	NCBI Databases Homepage	2	Université Laval York University
	Nucleic Acids Research (databases compilation)	2	Université de Montréal (Bio-informatiquepratique) Université de Montréal (Bio-informatiquethéorique)
	Nucleotide	2	McGill University Université Lava
	PROSITE	2	Université de Montréal (Bio-informatiquepratique) Université de Montréal (Bio-informatiquethéorique)
	Protein Information Resource	2	Université de Montréal (Bio-informatiquethéorique) York University
	Saccharomyces Genome Database	2	Université Laval Université de Montréal (Bio-informatiquepratique)
	Structure (Molecular Modeling Database)	2	Université Laval York University
	The Arabidopsis Information Resource (TAIR)	2	McGill University Université Laval
	WormBase	2	McGill University Université Laval
	Zebrafish Information Network (ZIN)	2	McGill University Université Laval
Tutorial	NCBI tutorials	4	McGill University Université de Montréal (Bio-informatiquepratique) Université de Montréal (Bio-informatiquethéorique) York University
Journal	Nucleic Acids Research (Web servers-specific issues. Note: different years)	4	McGill University Université de Montréal (Bio-informatiquepratique) Université de Montréal (Bio-informatiquethéorique) York University
	Cold Spring Harbor Protocols	3	McGill University Université Laval Université de Montréal (Bio-informatiquethéorique)
	Biochemistry	2	Université Laval Université de Montréal (Bio-informatiquethéorique)
	Bioinformatics	2	University of Manitoba Université de Montréal (Bio-informatiquethéorique)
	BMC bioinformatics	2	Université Laval University of Manitoba
	Nature	2	Université Laval Université de Montréal (Bio-informatiquethéorique)
	Nature Protocols	2	McGill University Université Laval
	Nucleic Acids Research	2	McGill University University of Manitoba
	Nucleic Acids Research (Databases-specific issues. Note: different years)	2	McGill University York University (with York listing two a link tothe 2017 issue and a table of the “golden set”, i.e., the most popular databases featured in multiple NAR issues)
	Science	2	Université Laval Université de Montréal (Bio-informatique théorique)
Repository	arXiv	2	McGill University Université Laval
Book	Dictionary of biomedicine	2	Simon Fraser University Université Laval
Citation index	Web of Science	2	McGill University Université Laval
Video	NLM YouTube channel	2	Université de Montréal (Bio-informatique pratique) York University
Website	Bioinformatics.ca	2	University of Calgary York University
	MEDLINE Plus: Genetics	2	York University Université de Montréal (Bio-informatique pratique)

Additionally, Table 3 summarizes the most commonly referenced journals across bioinformatics LibGuides. *Nucleic acids research* stands out as the most frequently linked journal, appearing in five different LibGuides across multiple institutions (McGill, Manitoba, UdeM, and York). This indicates its central importance to the bioinformatics field, particularly for its database and web server issues *. Cold Spring Harbor protocols* and *Nature* are the next most common journals, each appearing in three different LibGuides. The remaining journals (*Biochemistry, Bioinformatics, BMC bioinformatics, Nature protocols*, and *Science*) each appear in two different LibGuides. Of the 91 journals referenced throughout the LibGuides, most (n=68) are only recommended once. Laval and UdeM (Bio-informatique théorique) reference the widest variety of these 10 core journals in their LibGuides.

Table 4 describes the number of resource types across LibGuides. The most common resource type was database, making up 30.2% (n=171 items) of all resources. This was followed by journals at 16.1% (n=91), books at 14% (n=79), and websites at 12.2% (n=69). Notably, of the 79 books listed across 11 LibGuides, there was only one book, *Dictionary of biomedicine*, listed on two different LibGuides. Less common resource types include applications and tools at 5.8% (n=33), publishers at 5.5% (n=31), tutorials at 4.2% (n=24), videos at 3.4% (n=19), and repositories at 3.4% (n=19).

**Table 4:** Resource types by numbers and overall percentage of the total resources across all LibGuides (total of 566 resources)

Resource type	Quantity across guides	Overall percentage ofthe total resources across all LibGuides
Database	171	30.2%
Journal	91	16.1%
Book or ebook	79	14%
Website	69	12.2%
Application/tool	33	5.8%
Publisher	31	5.5%
Tutorial	24	4.2%
Repository	19	3.4%
Video	19	3.4%
Conference publication	10	1.8%
Citation index	8	1.4%
Internal resource (such as library homepage or link to a library PDF)	5	0.9%
Article	3	0.5%
Blog	3	0.5%
Newspaper article	1	0.2%

Table 5 describes the most common resource providers across LibGuides. Due to sheer volume, providers that were responsible for only one resource are not included in this table. NCBI is by far the most prominent resource provider, contributing 79 resources (14% of the total listed resources across all 11 LibGuides). This reflects NCBI’s central role in providing bioinformatics databases, tools, and information. Other major providers include Springer Nature with 5% of resources (n=28), internal resources with 3.9% (n=22), Elsevier and Wiley each with 3.5% (n=20) of resources, and the European Bioinformatics Institute (EBI), providing 3.2% (n=18) of resources. Table 5 shows a mix of major scientific publishers (e.g., Elsevier, Wiley, Springer Nature), specialized bioinformatics organizations (e.g., NCBI, EBI), academic institutions (e.g., MIT, McGill), as well as organism-specific databases (e.g., FlyBase, ZFIN, Saccharomyces Genome Database). Also represented, primarily due to York University’s LibGuide, are educational resources such as Khan Academy and Bozeman Science. Most providers (n=163) contributed less than 1% (one or two resources) across the LibGuides, indicating a diverse ecosystem of specialized resources in bioinformatics.

**Table 5:** Resource providers (n=68) of multiple resources by numbers and overall percentage of the total resources across all LibGuides. Note: providers of single resources are not counted in the table

Resource provider	Number of resources provided	Overall percentage ofthe total resources across all LibGuides
National Center for Biotechnology Information (NCBI)	79	14%
Springer Nature	28	5%
Internal resource(a link to either another page within the university or another LibGuide)	22	3.9%
Elsevier	20	3.5%
Wiley	20	3.5%
European Bioinformatics Institute	18	3.2%
Nature Publishing Group	15	2.7%
Nucleic Acids Research	13	2.3%
ExPasy	10	1.8%
National Library of Medicine	10	1.7%
Chemical Abstracts Service	9	1.6%
Cold Spring Harbor Laboratory	9	1.6%
CRC Press	9	1.6%
RCSB	8	1.4%
Khan Academy	7	1.2%
BioMed Central	6	1.1%
Massachusetts Institute of Technology	6	1.1%
UniProt	6	1.1%
RSC Publishing	6	1.1%
American Association for the Advancement of Science	5	0.9%
FlyBase	5	0.9%
Frontiers Media	5	0.9%
Oxford University Press	5	0.9%
Web of Science	5	0.9%
Wikipedia	5	0.9%
American Chemical Society	4	0.7%
Federation of European Biochemical Societies	4	0.7%
Federation of European Microbiological Societies	4	0.7%
Microbiology Society	4	0.7%
Portland Press	4	0.7%
Taylor & Francis	4	0.7%
Alliance for Genome Resources	3	0.5%
Bioinformatics.ca	3	0.5%
Bioinformatics.org	3	0.5%
Bozeman Science (YouTube channel)	3	0.5%
DeBoeck Supérieur	3	0.5%
Phoenix Bioinformatics	3	0.5%
National Human Genome Research Institute	3	0.5%
National Institutes of Health	3	0.5%
Open Bioinformatics Foundation	3	0.5%
Saccharomyces Genome Database	3	0.5%
Scitable by Nature Education	3	0.5%
ZFIN	3	0.5%
Academic Press	2	0.3%
American Society for Microbiology	2	0.3%
Applied Microbiology International	2	0.3%
arXiv	2	0.3%
BioStars.org	2	0.3%
Ciliates	2	0.3%
DDBJ	2	0.3%
Dunod	2	0.3%
eLife Sciences Publications	2	0.3%
Government of Alberta	2	0.3%
Humana Press	2	0.3%
Infrastructure Services for Open Access	2	0.3%
International Society for Computational Biology	2	0.3%
International Symposium on Bioinformatics Research and Applications	2	0.3%
Joint Genome Institute	2	0.3%
Mary Ann Liebert	2	0.3%
McGill University	2	0.3%
National Biomedical Research Foundation	2	0.3%
National Cancer Institute	2	0.3%
New England Biolabs	2	0.3%
Ovid	2	0.3%
ProQuest	2	0.3%
University of California Santa Cruz	2	0.3%
WellcomeTrust Sanger Institute	2	0.3%
Wolfram	2	0.3%

## Discussion

We contend that while there may not be an immediately identifiable overlap of bioinformatics resources on the LibGuides examined, the breadth of resources listed speaks to the interdisciplinarity of bioinformatics, offering a multitude of pathways for health librarians to approach the field. Through our analysis of the 11 bioinformatics LibGuides associated with Canadian academic libraries, we noticed a number of characteristics that warrant in-depth discussion for an LIS audience. Specifically, the variance in intended audience in these LibGuides, commonalities that *were* observed across the 11 LibGuides, the diversity of listed resources as a reflection of bioinformatics diversity as a field, and the American focus of the suggested resources. Taken together, these throughlines demonstrate what this diversity means for librarians and other information professionals using and teaching bioinformatics resources.

### Audience

The range of subject connections seen in the 11 bioinformatics LibGuides examined in this study reflect the field’s inherent interdisciplinary nature, suggesting that resource selection for bioinformatics LibGuides is influenced by the academic contexts. The LibGuides were part of basic medical sciences (Manitoba), biology (Winnipeg), microbiology and biochemistry (Laval), molecular biology and biochemistry (SFU), plant biology (Toronto), biochemistry and molecular medicine (UdeM practical), informatics (UdeM theoretical), and professional development (Calgary). As for York, McGill, and Guelph, they were categorized as solely being about bioinformatics.

Generally, the number of LibGuides lined up proportionally with the number (n=44) of graduate and undergraduate bioinformatics programs across Canada [18], which correlates with findings in the literature that roughly a quarter of librarians offered services like online LibGuides to disseminate bioinformatics resources [14]; while bioinformatics databases have been more recently addressed in the literature [19], the dissemination of bioinformatics resources in the Canadian university context remains understudied. Some of the universities, such as SFU and UdeM, had both a program and a LibGuide, while many others had bioinformatics programs but no guide. Bioinformatics programs often correlate with medical schools [14], however with only five universities having a LibGuide and medical school (Calgary, Laval, Manitoba, McGill, and UdeM) these LibGuides did not appear together.

There is some correlation of resources with the disciplinary background or subject responsibilities of the liaison librarians responsible for these LibGuides. An example of this is UdeM’s guides, both theoretical and practical, which were developed by different librarians. Its theoretical LibGuide is provided under the auspices of informatics, mathematics, and the natural sciences, and recommends books like *Algorithms in computational molecular biology* and *Bioinformatics algorithms*. Conversely, the UdeM practical LibGuide suggests various NCBI databases and books like *Yeast systems biology: methods and protocols* and *Progress in genomic medicine*. While the content of each guide aligns with its stated purpose, these also reflect the librarians’ subject responsibilities, with one falling on the side of health and medicine and the other on mathematics. Bioinformatics’ interdisciplinarity at the intersection of multiple sciences [1] translates into as many possible angles from which to approach it. In turn, this influences what resources are recommended to users via ready reference. This reflects previous literature on how scientists interact with and view bioinformatics changing according to discipline [20,21].

### Commonalities across LibGuides

While we found a set of bioinformatics resources that appear consistently across multiple university LibGuides, none pass over the line into majority representation. BLAST, Protein Data Bank, and PubMed each appear in five institutions’ LibGuides, suggesting their importance to the discipline. Another possibility is what they provide is unique enough that other resources do not cover the same content. For example, to look at gene and genomic data, there are a range of resources available, including Gene, Genome, Gene Expression Omnibus, Ensembl, among others. These being the most frequently referenced resources demonstrates the overwhelming representation of databases, constituting nearly 30% of all listed LibGuide resources. This highlights the data-intensive nature of bioinformatics across institutions.

Many of the LibGuides follow a pedagogical approach, highlighting tutorials for resources. This aligns with traditional uses of LibGuides to provide ready reference and help users navigate the vast amount of information [3]. This also demonstrates a lack of intuitiveness or ease of use for new users when it comes to bioinformatics tools and databases, something discussed in the literature [22–24]. Notably, the tutorials were present exclusively alongside databases, particularly specialized genome or bioinformatics-related databases. For example, Manitoba did not contain tutorials, focusing instead on the literature, nor did Calgary, which focused instead on job searching.

Among the most frequently recommended resources were specialized databases for different model organisms (i.e., FlyBase, WormBase, Saccharomyces Genome Database, etc.). Similarly, McGill, UdeM (Bio-informatique pratique), and York all had sections dedicated to model organisms. These mark the importance of model organisms to bioinformatics, which have dedicated research that produced completely sequenced genomes [25,26]. Model organisms help test bioinformatics tools and bioinformatics can in turn open up new avenues of interpretation for the vast amounts of data generated by model organism studies [27].

Giants across the science research landscape, such as PubMed, MEDLINE, and Web of Science, were well represented, if not only in numbers, then in design. Figure 1 shows their primacy by being the first option when users enter Laval’s LibGuide.

Figure 2 demonstrates Guelph’s sparse and to-the-point choice of resources. This is reflected in the literature on faculties’ regular use (daily or weekly) of bioinformatics resources [3,14], as well as another study that found PubMed was within the top ten most mentioned bioinformatics resources in the literature [19].

While we found several commonalities across LibGuides, they demonstrated thematic throughlines from best practices (i.e., importance of data, how to use resources, model organisms) rather than showing a clear and overwhelming overlap of commonly cited resources.

### Diversity of bioinformatics resources as a reflection of diversity of the discipline

There is notable diversity of resources across the LibGuides, collectively painting bioinformatics as a discipline that requires both domain-specific knowledge and familiarity with universal tools and databases. The majority of references appeared in only one guide; similarly, most resource providers only contribute single resources to the research guides. The variety of resources highlighted in the consulted LibGuides indicates the presence of information professional curation in such a way as to reflect the particular institution’s needs. Notably, “a subject guide is not a laundry list of every reference book or Internet link related to a topic” [28], but rather a curated document of relevant sources. As such, it follows that not all resources will appear in all LibGuides consulted for this project. Yet, the sheer diversity of materials available reflects the ever-growing plethora of bioinformatics resources.

While databases dominate as the most recommended resource type, 84 out of the 171 databases only appear once. This means 32 databases appear more than once, accounting for the other 87 databases. This indicates that though there is significant specialization in the field, there are also a variety of options available to support research and study. For example, both McGill’s LibGuide and UdeM’s Bioinformatique pratique LibGuide have subsections on model organisms and proteins. However, the overlap of sources is limited to three common proteomic resources and two for model organisms out of a wider range of resources. Both guides also provide different protein visualization resources.

As for journals, the overwhelming majority are only referenced once across 11 LibGuides. The listed journals span biochemistry, molecular biology, and computational resources, with a mix of specialized bioinformatics journals and broader scientific publications like *Nature* and *Science*.

The distribution of resource types reflects the nature of bioinformatics as a field heavily dependent on databases, with a significant reliance on digital resources (websites, applications, and tutorials) alongside traditional academic publications. While NCBI dominates, most providers (n=154) contributed less than 1% (one or two resources) across the LibGuides, indicating a diverse ecosystem of specialized resources in bioinformatics.

### American focus

The geographic and funding concentration of bioinformatics LibGuides resources reveals a vulnerability. There are many directions and approaches to bioinformatics, which the 11 guides illustrate. Yet, as demonstrated by the primacy of NCBI, overwhelmingly the references are provided by American institutions, often via American funding. (When considering the broader organization, namely NLM and NIH, this coverage increases slightly from 14% to 16% of resources.) Given the current political landscape [29,30], this raises concerns around both access to and veracity of data that have long been taken for granted. Regardless, the thread of American funding and institutions supporting this work represents a concern for the field. While this can be taken as one microcosm of current anti-science policies, it also demonstrates a clear downside of the historical anglocentric dominance among citation indexes and metrics [31,32]. Bibliometric studies have found some increased geographical representation in recent years in medical publishing for example, however, the United States, and the United Kingdom continue to dominate [31,33].

### Limitations

As with any research, ours has several limitations. Firstly, the search strategy may have missed some LibGuides, including guides not hosted by SpringShare. Additionally, relevant material might be included in guides under other subjects (i.e., genetics, biochemistry, molecular biology, or others), without being explicitly titled bioinformatics. While LibGuides can tell us about the recommended sources, they do not tell us much about usage. As well, we mainly provided quantitative assessment and were limited in our qualitative assessment, while prior research of guides has included surveys to address these two points. These would be excellent avenues for new research.

### Conclusion

From its earliest iterations in the 1960s to the present day, bioinformatics remains very fluid; this fluidity extends to the resources researchers may consult. As these 11 LibGuides demonstrate, there is a wide variety of resources that are available to bioinformatics researchers—but what they find depends on where they choose to look. Put another way, a researcher perusing Guelph’s Bioinformatics LibGuide may encounter a different selection of resources than a researcher searching McGill’s LibGuide on the same subject. Even looking more specifically at topics like proteins or model organisms could lead users to different resources. The wide range of possible approaches to bioinformatics, as the LibGuides demonstrate, offers a variety of starting points for novices to the bioinformatics field and its plethora of resources. It also provides a rich, generous pool in which the biological researcher may swim. However, its suggestion of a field in constant flux means someone newly entering it may be overwhelmed by the vast number of resources available—and, in fact, may not find the information they are looking for. This fluidity can create confusion about what is considered a bioinformatics resource.

The reliance on American-funded resources in bioinformatics LibGuides highlights a pressing need for academic librarians to advocate for more diverse resource selections and cultivate relationships with international data repositories. This type of effort is one practical safeguard against political vulnerabilities in scientific information access. It would also represent an opportunity for libraries to advance more equitable scholarly communication practices within interdisciplinary scientific fields.

Through our analysis of these 11 LibGuides associated with Canadian university libraries, we explored the diversity of resources available to the bioinformatic researcher—and how those resources may be curated by librarians. The current American focus of the collected resources offers one particular viewpoint of bioinformatics, even as the resources themselves are distinct. These distinct resources reflect the diverse origins of bioinformatics tools and knowledge. It also reflects the field’s interdisciplinary nature, drawing from multiple scientific domains and requiring various specific tools and knowledge sources, which the information professional is equipped to curate, including in asynchronous platforms such as LibGuides.

## Footnotes

2 There were an additional 18 resources that were broken links.
